# Stapler insertion angle toward the esophagus reduces the incidence of early postoperative Roux stasis syndrome after distal gastrectomy in minimally invasive surgery

**DOI:** 10.1186/s12893-023-01954-3

**Published:** 2023-03-11

**Authors:** Kozo Yoshikawa, Mitsuo Shimada, Takuya Tokunaga, Toshihiro Nakao, Masaaki Nishi, Chie Takasu, Hideya Kashihara, Yuma Wada, Toshiaki Yoshimoto

**Affiliations:** The Department of Surgery, The University of Tokushima, 3-18-15 Kuramoto-cho, Kuramoto-Cho, Tokushima, 770-8503 Japan

**Keywords:** Rous stasis syndrome, Distal gastrectomy, Stapler insertion angle

## Abstract

**Background:**

Roux stasis syndrome (RSS) after Roux-en-Y (RY) reconstruction significantly prolongs the hospital stay and decreases the quality of life. The purpose of the present study was to evaluate the incidence of RSS in patients who underwent distal gastrectomy for gastric cancer and to identify the factors related to the development of RSS after mechanical RY reconstruction in minimally invasive surgery (MIS).

**Methods:**

This study included 134 patients who underwent distal gastrectomy in MIS with mechanical RY anastomosis. RSS was defined as the presence of symptoms such as nausea, vomiting, or abdominal fullness, and the confirmation of delayed gastric emptying on imaging or gastrointestinal fiber testing. Clinical data were checked, including body mass index, operative procedure, age, sex, operative time, blood loss volume, extent of lymph node dissection, final stage, stapler insertion angle, method of entry hole closure. The relationship between the incidence of RSS and these factors was analyzed.

**Results:**

RSS occurred in 24 of 134 patients (17.9%). RSS occurred significantly more frequently in patients with D2 lymphadenectomy than in patients with D1 + lymphadenectomy (p = 0.04). All patients underwent side-to-side anastomosis via the antecolic route. The incidence of RSS was significantly greater in patients with a stapler insertion angle toward the greater curvature (n = 20, 22.5%) versus the esophagus (n = 4, 8.9%) (p = 0.04). The multivariate logistic regression model revealed that the stapler insertion angle to the greater curvature is identified as independent risk factor for RSS (OR 3.23, 95%Cl 1.01–10.3, p = 0.04).

**Conclusion:**

Stapler insertion angle toward the esophagus may reduce the incidence of early postoperative RSS rather than toward the greater curvature.

## Introduction

The three main reconstruction procedures after distal gastrectomy are Billroth I and II and Roux-en-Y (RY) reconstruction. Although the best reconstructive procedure after distal gastrectomy remains controversial, RY reconstruction has many advantages over the Billroth procedures. Compared with Billroth I and II reconstructions, RY reconstruction prevents bile reflux into the gastric remnant, prevents gastritis, and has decreased incidences of gastric cancer recurrence and postoperative complications including anastomotic leakage [1]. However, delayed gastric emptying, known as Roux stasis syndrome (RSS), occurs more frequently after RY reconstruction [2].

RSS occurs in 10–30% of patients after RY reconstruction [3–5], and causes symptoms such as abdominal fullness, abdominal pain, nausea, and vomiting without mechanical obstruction. RSS significantly prolongs the hospital stay and decreases the quality of life. Many etiologies of RSS have been reported, including an aberrant propagation of the migrating motor complex in the Roux limb, the length of the Roux limb [6, 7], and the effects of an ectopic pacemaker arising in the Roux limb that drives contractions in reverse or oral direction toward the stomach [8]. However, the definitive factors related to the occurrence of RSS remain unclarified.

Previous study has compared the incidence of RSS among patients who underwent RY reconstruction via the hand-sewn technique versus the mechanical technique [6]. However, there are few studies that focused on the incidence of RSS in patients who underwent mechanical RY anastomosis in minimally invasive surgery (MIS), including laparoscopic and robotic surgery.

Previous reports suggested that the straight anastomotic shape in the post-operative contrast gastrography can reduce the RSS [9]. So we hypothesize that stapling to the esophagus makes a smooth passage and reduces the incidence of RSS.

The purpose of the present study was to evaluate the incidence of RSS in patients who underwent distal gastrectomy for gastric cancer and to identify the factors related to the development of RSS after mechanical RY reconstruction in MIS.

## Patients and methods

Between January 2007 and April 2021, 151 patients underwent distal gastrectomy with RY anastomosis in MIS. After the exclusion of 17 patients who underwent hand-sewn anastomosis, the study included 134 patients who underwent distal gastrectomy in MIS with mechanical RY anastomosis.

### Methods

Clinical and patient data were checked, including body mass index, operative procedure, age, sex, operative time, blood loss volume, extent of lymph node dissection, final stage, stapler insertion angle (toward the greater curvature or the esophagus), method of entry hole closure (directly through laparotomy, intracorporeal stapling, or intracorporeal hand-sewing). The relationship between the incidence of RSS and these factors was analyzed.

RSS was defined as the presence of symptoms such as nausea, vomiting, or abdominal fullness, and the confirmation of delayed gastric emptying on imaging. In detail, contrast agent doesn’t pass through the anastomosis site within about 5 min after swallowing the contrast agent. Patients diagnosed with postoperative ileus or anastomotic stricture through radiologic examinations, such as abdominal radiography or upper gastrointestinal series radiography, were not considered to have RSS. The surgeons checked each patient for symptoms of RSS daily. Patients with symptoms of RSS had their oral intake stopped until nausea or vomiting had completely resolved.

### Distal gastrectomy procedure

Even the tumor is in L-region. Two-thirds of the stomach was resected. And when the estimated remnant stomach is only the fundus. total gastrectomy was performed in our institution. A vagus-sparing gastrectomy was not performed.

### Anastomotic procedure

**Fig. 1:**
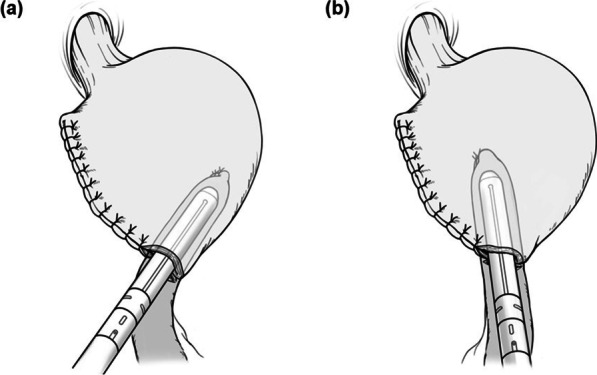
Gastrojejunostomy is performed. **a** The stapler was inserted toward the greater curvature. **b** The stapler was inserted toward the esophagus

The jejunum was transected about 20 cm from the Treitz ligament and brought through the antecolic route after the division of the jejunum. The distance between the gastrojejunostomy (GJ) and the jejunojejunal anastomosis was about 40 cm. Laparoscopic linear staplers were used for all GJ and jejunojejunal anastomoses, with the common entry holes closed by hand-sewing or stapling. All reconstructions performed before 2012 were completed extracorporeally, while subsequent reconstructions were completed using extracorporeal or intracorporeal anastomotic methods. GJ was performed using one of two stapler insertion angles. The stapler was either inserted toward the greater curvature (Fig. 1a) or the esophagus (Fig. 1b); regardless of the stapler insertion angle, all anastomoses were performed using the side-to-side technique. 60 mm stapler was used for anastomosis in all cases. Mesenteric defects and Petersen's space were closed with continuous non-absorbable sutures.

### Postoperative protocol

Patients were allowed to drink water on POD 1, and take a nutritional supplement on POD 2, POD 3. they could begin eating soft foods on POD 4, with a more solid diet served each subsequent day.

### Statistical analyses

Data were analyzed using the JMP statistical software program (SAS Institute Inc., Cary, NC, USA). The χ^2^ test or Fisher’s exact test was used to compare categorical variables. The Mann–Whitney U test was used to compare continuous variables. Quantitative variables are presented as the mean ± standard deviation. The variables for which P value were less than 0.1 in univariate analysis were included in multivariate analysis. P values of < 0.05 were considered statistically significant.

## Results

RSS occurred in 24 of 134 patients (17.9%). There were no postoperative in-hospital deaths. Follow-up period of the early RSS is at least the 6 months. The incidence of RSS was not significantly associated with body mass index, sex, age, operation approach, operative time, blood loss volume, or tumor stage. However, RSS occurred significantly more frequently in patients with D2 lymphadenectomy than in patients with D1 + lymphadenectomy (p = 0.04) (Table 1). One patient had anastomotic leakage; this was managed via conservative treatment.

**Table 1:** Patient’s characteristics

	RSS(−) (n = 110)	RSS(+) (n = 24)	p value
Sex			
Male	69	13	0.43
Female	41	11	
Age	63.9 ± 10.8	67.2 ± 8.6	0.16
BMI (kg/m^2^)	22.7 ± 3.2	22.7 ± 3.2	0.94
Operative approach			
Laparoscopic	107	21	0.06
Robotic	3	3	
LN dissection			
D1 +	70	10	0.04
D2	40	14	
Operation time	338 ± 57.5	358 ± 80.0	0.17
Blood loss	49.1 ± 65.1	76.7 ± 117.7	0.11
Stage			
I	87	17	0.28
II	19	4	
III	4	3	

*RSS* Roux stasis syndrome, *BMI* body mass index, *LN* lymph node

All patients underwent side-to-side anastomosis via the antecolic route. Gastrojejunostomy was performed in direct procedure in 112 patients and laparoscopic procedure in 22 patients. Stapler was inserted toward the esophagus in 45 patients and toward the greater curvature in 89 patients. The incidence of RSS was significantly greater in patients with a stapler insertion angle toward the greater curvature (n = 20, 22.5%) versus the esophagus (n = 4, 8.9%) (p = 0.04). The incidence of RSS was not significantly affected by the entry hole closure method (directly through a laparotomy (16.5%), intracorporeal stapling (16.7%), intracorporeal hand-sewing (42.8%)) (Table 2).

**Table 2:** Patient’s characteristics concerning the anastomosis

	RSS (−) (n = 110)	RSS (+) (n = 24)	p value
Gastrojejunostomy			
Direct procedure	92	20	0.97
Laparoscopic procedure	18	4	
Insertion angle			
Greater curvature	69	20	0.04
Esophagus	41	4	
Entry hole closure			
Direct from laparotomy	101	20	0.28
Intracorporeal stapler	5	1	
Intracorporeal hand sawn	4	3	

*RSS* Roux stasis syndrome

Univariate analysis revealed that RSS was significantly associated with D2 lymphadenectomy and stapler insertion angle to the greater curvature. Multivariate logistic regression model revealed that the stapler insertion angle to the greater curvature is identified as independent risk factor for RSS (OR 3.23, 95%Cl 1.01–10.3, p = 0.04). (Table 3).

**Table 3:** Univariate and multivariate analyses of risk factors associated with RSS

	Univariate analysis			Multivariate analysis		
	OR	P value	95% CI	OR	P value	95% CI
Female	1.42	0.43	0.58–3.45			
Age > 75	1.09	0.88	0.33–3.60			
BMI < 25	1.71	0.36	0.53–5.42			
Laparoscopic approach	5.09	0.06	0.96–26.9	3.12	0.20	0.52–18.4
D2 lymphadenectomy	2.45	0.04	0.99–6.02	1.90	0.18	0.72–4.95
Operation time > 332 min	1.71	0.23	0.70–4.19			
Blood loss > 54 ml	1.14	0.77	0.43–3.02			
Insertion angle (GC)	2.97	0.04	0.94–9.29	3.23	0.04	1.01–10.3

*BMI* body mass index, *GC* greater curvature

Table 4 shows the data of the patients with RSS (n = 24). Nasogastric tube (NG) re-insertion was performed in 17 patients. The mean onset of RSS was 10.5 ± 4.5 days after surgery. The fasting period was significantly shorter in patients who did not have the NG re-inserted (4.4 ± 1.6 days) compared with those who did have the NG re-inserted (10.2 ± 1.1 days, p < 0.01).

**Table 4:** Patient’s characteristics in RSS group

Number	Angle	Age	Sex	POD of RSS (day)	NG tube insertion	NG tube insertion period (day)	Fast period (day)
1	Greater curvature	57	F	11	−	−	1
2	Greater curvature	71	F	10	+	8	14
3	Greater curvature	64	M	8	−	−	5
4	Greater curvature	60	M	9	−	3	7
5	Greater curvature	73	F	10	+	5	5
6	Greater curvature	61	M	20	−	−	7
7	Greater curvature	64	F	12	+	1	4
8	Greater curvature	57	M	13	+	14	18
9	Greater curvature	62	F	10	+	11	13
10	Greater curvature	53	M	12	+	7	8
11	Greater curvature	70	F	8	−	−	1
12	Greater curvature	71	F	9	+	1	5
13	Greater curvature	61	M	15	+	7	12
14	Greater curvature	70	M	4	+	6	14
15	Greater curvature	68	F	8	−	−	4
16	Greater curvature	58	M	7	−	−	6
17	Greater curvature	63	M	2	+	3	5
18	Greater curvature	85	M	10	+	6	10
19	Greater curvature	79	M	8	+	3	5
20	Greater curvature	71	M	23	+	4	13
21	Esophagus	72	F	15	+	6	10
22	Esophagus	61	F	9	+	6	9
23	Esophagus	81	F	9	+	5	7
24	Esophagus	83	M	11	+	7	21

*RSS* Roux stasis syndrome, *POD* post operative day, *NG* nasogastric

## Discussion

The present study showed that a stapler insertion angle toward the esophagus is associated with a significantly lower incidence of RSS than a stapler insertion angle toward the greater curvature, while D2 lymphadenectomy is associated with a significantly higher incidence of RSS than D1 + lymphadenectomy. RSS was not significantly associated with other factors, including sex, age, operative time, and blood loss volume. No previous study has reported the relationship between the incidence of RSS and the stapler insertion angle for mechanical RY reconstruction in MIS.

The RY procedure is reportedly associated with a lower incidence of postoperative complications, including anastomotic leakage, and improved postoperative quality of life [1, 10–12]. However, other studies have suggested that RY reconstruction has limited advantages compared with Billroth I reconstruction because RY reconstruction frequently induces RSS, causing longer postoperative hospitalization [13, 14], and RY increases the risk of internal hernia because of the need for two anastomoses and one duodenal stump closure [15]. Furthermore, RY reconstruction may cause problems if endoscopic retrograde cholangiopancreatography is necessary for the diagnosis and treatment of pancreaticobiliary disease after distal gastrectomy [16].

The symptoms of gastric retention, such as abdominal pain, nausea, vomiting, abdominal distention, and loss of appetite, occur in approximately 10%–30% of patients who undergo RY reconstruction after distal gastrectomy [3–5]. RSS is characterized by symptoms of upper gut stasis after RY reconstruction. The factors causing RSS after distal gastrectomy are remnant gastric atony and stasis of the Roux limb itself. Vagotomy can affect remnant gastric atony, but there are several reasons for the stasis of the Roux limb [17]. First, RSS is caused by the separation of the Roux limb from the natural pacemaker of the small bowel, which is located in the proximal duodenum [8]. An ectopic pacemaker induces retrograde constriction in the proximal region [18]. Roux limb movement and occasionally reversed peristalsis then delay the passage of gastric contents. Second, the length of the Roux limb is correlated with the RSS. A Roux limb length of longer than 40 cm reportedly increases the incidence of RSS [7]. Although all patients in the present study had a Roux limb length of approximately 40 cm and cut the intestine completely, some patients still developed postoperative RSS, suggesting that other factors affected the incidence of RSS. Therefore, we focused on the surgical technique. Although the entry hole closure method did not affect the incidence of RSS, the stapler insertion angle was identified as a predictive factor. Third, RSS may be related to the Roux limb location. In pylorus-preserving pancreaticoduodenectomy, antecolic reconstruction of the duodenojejunostomy decreases the incidence of delayed gastric emptying compared with retrocolic reconstruction [5], because the location of the remnant stomach in antecolic reconstruction is parallel to the long axis of the body, allowing for the smooth passage of food [19]. Moreover, a straight anastomotic shape on contrast radiography is related to a lower incidence of RSS [9]. Inserting the stapler toward the esophagus achieves an ideal straight anastomotic passage, which reduces the incidence of RSS. As the elevated portion of the small intestine is parallel to the long axis of the remnant stomach, the position of the Roux limb affects the incidence of RSS.

In mechanical anastomosis, the stapler is inserted into the small intestine. Although the insertion entry hole is made after pre-checking the stapler length, it is impossible to perfectly align the position of the hole. Therefore, there is a resultant space, and the intestine tends to decline. We speculate that RSS results from the edge of the elevated portion of the small intestine being declined and the anastomotic site is not straight if there is a free space. However, in the patients with a stapler insertion angle toward the esophagus, there was no space in the posterior portion of the remnant stomach, and it prevented the rotation of the Roux limb.

Although RSS is reported in Roux-en-Y gastric bypass (RYGB), the incidence of RSS is not nearly as high as it is after oncologic resection [20]. The small pouch in the upper stomach was used for anastomosis with jejunum. Due to the small stomach, stapler was inserted toward the esophagus. This matter may be related to the low incidence of RSS after RYGB.

In this study, all patients underwent about two-thirds gastrectomy. Large remnant stomach related with the RSS in previous report [21]. So we should not preserve the large remnant stomach.

## Limitations

This study has limitations. First, the sample size was small. Second, the study had a retrospective design and included two types of GJ (direct and intracorporeal).

## Conclusions

The stapler insertion angle may reduce the incidence of early RSS. The incidence of early RSS was significantly lesser in patients with a stapler insertion angle toward the esophagus rather than toward the greater curvature.

## Data Availability

The datasets used during the current study are available from the corresponding author on reasonable request.

## References

[1] Lee MS, Ahn SH, Lee JH, Park DJ, Lee HJ, Kim HH (2012). What is the best reconstruction method after distal gastrectomy for gastric cancer?. Surg Endosc.

[2] Hoya Y, Mitsumori N, Yanaga K (2009). The advantages and disadvantages of a Roux-en-Y reconstruction after a distal gastrectomy for gastric cancer. Surg Today.

[3] Gustavsson S, Ilstrup DM, Morrison P, Kelly KA (1988). Roux-Y stasis syndrome after gastrectomy. Am J Surg.

[4] Nakamura M, Nakamori M, Ojima T, Iwahashi M, Horiuchi T, Kobayashi Y (2016). Randomized clinical trial comparing long-term quality of life for Billroth I versus Roux-en-Y reconstruction after distal gastrectomy for gastric cancer. Br J Surg.

[5] Imamura H, Takiguchi S, Yamamoto K, Hirao M, Fujita J, Miyashiro I (2012). Morbidity and mortality results from a prospective randomized controlled trial comparing Billroth I and Roux-en-Y reconstructive procedures after distal gastrectomy for gastric cancer. World J Surg.

[6] Fujita T, Katai H, Morita S, Saka M, Fukagawa T, Sano T (2010). Short-term outcomes of Roux-en-Y stapled anastomosis after distal gastrectomy for gastric adenocarcinoma. J Gastrointest Surg.

[7] Le Blanc-Louvry I, Ducrotté P, Lemeland JF, Metayer J, Denis P, Ténière P (1999). Motility in the Roux-Y limb after distal gastrectomy: relation to the length of the limb and the afferent duodenojejunal segment—an experimental study. Neurogastroenterol Motil.

[8] Uyama I, Sakurai Y, Komori Y, Nakamura Y, Syoji M, Tonomura S (2005). Laparoscopy-assisted uncut Roux-en-Y operation after distal gastrectomy for gastric cancer. Gastric Cancer.

[9] Masui T, Kubora T, Nakanishi Y, Aoki K, Sugimoto S, Takamura M (2012). The flow angle beneath the gastrojejunostomy predicts delayed gastric emptying in Roux-en-Y reconstruction after distal gastrectomy. Gastric Cancer.

[10] Yang K, Zhang WH, Liu K, Chen XZ, Zhou ZG, Hu JK (2017). Comparison of quality of life between Billroth-I and Roux-en-Y anastomosis after distal gastrectomy for gastric cancer: a randomized controlled trial. Sci Rep.

[11] Nunobe S, Okaro A, Sasako M, Saka M, Fukagawa T, Katai H (2007). Billroth 1 versus Roux-en-Y reconstructions: a quality-of-life survey at 5 years. Int J Clin Oncol.

[12] Kojima K, Yamada H, Inokuchi M, Kawano T, Sugihara K (2008). A comparison of Roux-en-Y and Billroth-I reconstruction after laparoscopy-assisted distal gastrectomy. Ann Surg.

[13] Takiguchi S, Yamamoto K, Hirao M, Imamura H, Fujita J, Yano M (2012). A comparison of postoperative quality of life and dysfunction after Billroth I and Roux-en-Y reconstruction following distal gastrectomy for gastric cancer: results from a multi-institutional RCT. Gastric Cancer.

[14] Ishikawa M, Kitayama J, Kaizaki S, Nakayama H, Ishigami H, Fujii S (2005). Prospective randomized trial comparing Billroth I and Roux-en-Y procedures after distal gastrectomy for gastric carcinoma. World J Surg.

[15] Kitagami H, Morimoto M, Nozawa M, Nakamura K, Tanimura S, Murakawa K (2014). Evaluation of the delta-shaped anastomosis in laparoscopic distal gastrectomy: midterm results of a comparison with Roux-en-Y anastomosis. Surg Endosc.

[16] Namikawa T, Kitagawa H, Okabayashi T, Sugimoto T, Kobayashi M, Hanazaki K (2010). Roux-en-Y reconstruction is superior to billroth I reconstruction in reducing reflux esophagitis after distal gastrectomy: special relationship with the angle of his. World J Surg.

[17] Park JY, Kim YJ (2014). Uncut Roux-en-Y reconstruction after laparoscopic distal gastrectomy can be a favorable method in terms of gastritis, bile reflux, and gastric residue. J Gastric Cancer.

[18] Park YS, Shin DJ, Son SY, Kim KH, Park DJ, Ahn SH (2018). Roux stasis syndrome and gastric food stasis after laparoscopic distal gastrectomy with uncut Roux-en-Y reconstruction in gastric cancer patients: a propensity score matching analysis. World J Surg.

[19] Otsuka R, Natsume T, Maruyama T, Tanaka H, Matsuzaki H (2015). Antecolic reconstruction is a predictor of the occurrence of roux stasis syndrome after distal gastrectomy. J Gastrointest Surg.

[20] Bradley JF, Ross SW, Christmas AB, Fischer PE, Sachdev G, Heniford BT, Sing RF (2015). Complications of bariatric surgery: the acute care surgeon's experience. Am J Surg.

[21] Mukoyama T, Kanaji S, Sawada R, Harada H, Urakawa N, Goto H (2022). Assessment of risk factors for delayed gastric emptying after distal gastrectomy for gastric cancer. Sci Rep.

